# Exceedingly Higher co-loading of Curcumin and Paclitaxel onto Polymer-functionalized Reduced Graphene Oxide for Highly Potent Synergistic Anticancer Treatment

**DOI:** 10.1038/srep32808

**Published:** 2016-09-06

**Authors:** Kasturi Muthoosamy, Ibrahim Babangida Abubakar, Renu Geetha Bai, Hwei-San Loh, Sivakumar Manickam

**Affiliations:** 1 Centre for Nanotechnology and Advanced Materials (CENTAM), Faculty of Engineering, University of Nottingham Malaysia Campus (UNMC), Semenyih, Selangor 43500, Malaysia; 2 School of Biosciences, Faculty of Science, UNMC, Semenyih, Selangor 43500, Malaysia; 3 Biotechnology Research Centre, UNMC, Semenyih, Selangor 43500, Malaysia

## Abstract

Metastasis of lung carcinoma to breast and vice versa accounts for one of the vast majority of cancer deaths. Synergistic treatments are proven to be the effective method to inhibit malignant cell proliferation. It is highly advantageous to use the minimum amount of a potent toxic drug, such as paclitaxel (Ptx) in ng/ml together with a natural and safe anticancer drug, curcumin (Cur) to reduce the systemic toxicity. However, both Cur and Ptx suffer from poor bioavailability. Herein, a drug delivery cargo was engineered by functionalizing reduced graphene oxide (G) with an amphiphilic polymer, PF-127 (P) by hydrophobic assembly. The drugs were loaded via pi-pi interactions, resulting in a nano-sized GP-Cur-Ptx of 140 nm. A remarkably high Cur loading of 678 wt.% was achieved, the highest thus far compared to any other Cur nanoformulations. Based on cell proliferation assay, GP-Cur-Ptx is a synergistic treatment (CI < 1) and is highly potent towards lung, A549 (IC_50_ = 13.24 μg/ml) and breast, MDA-MB-231 (IC_50_ = 1.450 μg/ml) cancer cells. These positive findings are further confirmed by increased reactive oxygen species, mitochondrial membrane potential depletion and cell apoptosis. The same dose treated on normal MRC-5 cells shows that the system is biocompatible and cancerous cell-specific.

Metastasis of lung cancer cells to a secondary distant site such as breast and vice versa, remains a great challenge in cancer therapy^1^. It was reported that more than 80% of patients diagnosed with lung cancer is suffering from metastatic diseases^2^. Although chemotherapy may suppress and prevent the spread of cancer cells, its acute toxicity coupled with poor solubility, adverse side-effects^3^ as well as drug resistance in tumors^4^ dispute its usage and remain a concern in the medical field. Combination therapy with the usage of a natural and pharmacologically safe anticancer drug together with a highly potent yet toxic commercial-anticancer agent is an attractive approach to address these limitations. Natural anticancer drug has significantly lower toxicity, safe and easily available.

Curcumin (Cur) is one of such products. This yellow colored naturally occurring polyphenolic phytoconstituent is purified from the rhizome of the plant, *Curcuma longa*. Cur, despite being well-known for its anti-inflammatory, antioxidant and antibacterial properties, it is also an anticancer agent which was reported to be able to suppress and treat various types of malignancies^3^,^5^. Taxol or Paclitaxel (Ptx), on the other hand is a highly potent anticancer drug that is commercially available. Ptx has often been studied in conjunction with other chemotherapeutic agents to enhance its therapeutic effectiveness and to reduce its toxicity. It was reported to be able to inhibit cancer metastasis, however chemoresistance was also observed in some instances^6^.

Combination of Cur and Ptx is an attractive anticancer drug therapy. At mechanistic level, Ptx is a potent microtubule-stabilizing agent that triggers cell cycle arrest^7^, whereas Cur attacks biologically by regulating multiple signal transduction pathways^8^. By co-delivery, both of these drugs enhance caspase-3/7 activity, thus significantly increase apoptosis and inhibit lung and breast cancer metastasis^9^.

Despite these good therapeutic effects, Ptx and Cur however are hydrophobic. Cur has an extremely short biological half-life, slow dissolution rate and thus poor bioavailability^3^. Demand for effective Cur delivery strategies resulted into attractive systems for Cur formulations such as liposomes, polymeric micelles and polymeric nanoparticles, however, the drug loading capacity achieved was considerably low, i.e. only 15–20%^5^.

Graphene, the 2-D honeycomb lattice can be effectively utilized to impart solubility as well as a drug delivery agent. Extensive research has been carried out on graphene oxide (GO) as a loading system for anticancer drugs such as Ptx, doxorubicin and camptothecin^10^. However, most of the routes for synthesizing GO involve strong oxidizing agents, which are ultimately carried forward to the end-product. Moreover, GO is highly acidic which could cause damage to the normal cells, thus jeopardizing its role as a carrier in drug delivery system.

In this study, reduced graphene oxide (G) is used as a cargo system, instead of the conventional GO. Based on our previous report, the synthesized G is highly biocompatible towards normal cells, thus rendered suitable for drug delivery purposes^11^. With simple functionalization of G with an amphiphilic triblock co-polymer such as PF-127 (P), enhanced stability and solubility is expected whereby the polypropylene oxide (PPO) groups of polymer will be adsorbed on the surface of G via hydrophobic interaction, whereas the polyethylene oxide (PEO) brushes will extend out and impart solubility. The hydrophobic drugs, Cur and Ptx could then be loaded onto the empty spaces on G via pi-pi interactions between the drugs and the aromatic structures of G (Supplementary Fig. S1).

In this engineered design (Fig. 1), it is hypothesized that apart from enhanced solubility of Ptx and Cur, exceedingly high drug loading can be achieved. The vast unhindered surface on G would allow effective and maximal drug loading, which would otherwise be detrimental in GO due to the interference of epoxide functional groups on its surface. There are many reports on the combined drug regimen using Cur and Ptx, however the dose tested was often random, sometimes in the ratio of 1:1, contradicting with the main aim of reducing systemic toxicity. An attempt to use the lowest possible amount of Ptx, such as at the amount of sub-inhibitory concentration of 20% or IC_20_ along with sub-effective dose of IC_50_ of Cur has never been tested. In addition, the polymer-functionalized GP cargo is hypothesized to be non-toxic to normal cells and thus this nano hybrid material is expected to contribute a better drug delivery with increased effectiveness on cancer cell inhibition as compared to treatments with single drug agents. Thus, the current research focuses mainly on synthesis and characterization of the hybrid GP-Cur-Ptx drug delivery system along with biological assays to assertion the synergistic and anti-proliferative effects of Cur and Ptx loaded GP in treatment of breast and lung cancer.

## Results

### Chemical characterization of GP and drug loaded GP

**Figure 2:**
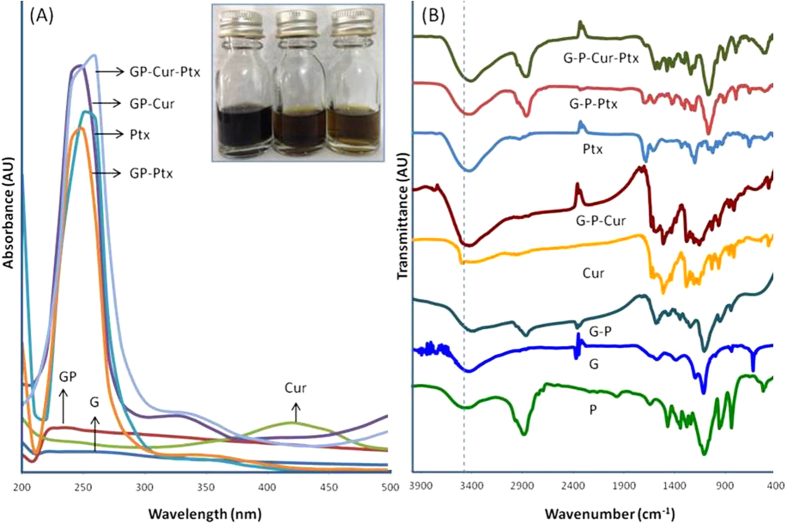
UV-Vis and FTIR spectra of drug loaded GP cargo. (**A**) UV-Vis spectra of Cur and Ptx loaded onto GP. Inset: Image of GP (left), GP-Cur (middle) and GP-Cur-Ptx (right). (**B**) FTIR spectra of Cur and Ptx loaded onto GP.

The black solution of GP turned into a yellowish-black upon loading of Cur and correspondingly appears faded with the addition of Ptx (Fig. 2A, inset). The successful loading of drugs onto GP was further confirmed by UV-Vis spectroscopy (Fig. 2A). The broad absorption peak at 250–260 nm is relative to G, which is due to the excitation of pi-plasmon of the graphitic structure^12^. Upon functionalization with polymer, the GP has an additional peak at 230 nm. Cur has a characteristic peak at 420 nm and a broad band ranged from 500–800 nm, which was also observed in GP-Cur and GP-Cur-Ptx. Likewise, the Ptx has a characteristic peak at 250 nm, which was also observed in GP-Ptx. Upon loading of Cur and Ptx onto the GP cargo, the characteristic peaks of Ptx and Cur appears red-shifted. Generally the red shift of absorption spectrum indicates an increase in the chain length of the double bond conjugation, which translates to successful drug loading and electron transfer^13^.

To further ascertain the successful conjugation between the drugs and the carrier, FTIR analysis was conducted on the modified G (Fig. 2B). In G, the appearance of a broad ν_C-O_ band at 1186–1112 cm^−1^ is due to the presence of residual oxygen atoms or cyclic ether accumulated at the edges of G after the reduction from GO^11^. Additionally, the peaks at 1593 and 1383 cm^−1^ represent the skeletal vibration of graphene sheets^14^. The polymer PF-127 has several characteristic peaks, specifically at 2954 and 2870 cm^−1^ representing ν_C-H_ from CH_3_ and CH_2_, respectively^15^. The presence of these characteristic peaks in GP confirms the functionalization of G with the polymer.

Two hydroxyl peaks of Cur were observed at 3509 and 3360 cm^−1^, which are due to free OH-groups of phenol and alcohol, respectively^16^. Other characteristic Cur bands were observed (Supplementary Table S1) and upon loading onto GP, the GP-Cur hybrid system exhibited similar Cur absorption peaks and appears slightly red-shifted, which validates the successful loading of Cur.

In Ptx, several characteristic peaks were observed at 3476, 1728 and 1170–1070 cm^−1^, which relate to ν_N-H_, ν_C=O_ and ν_C-O_ stretching bands, respectively^17^. Similarly, upon loading onto GP the appearance of these Ptx peaks in the GP-Ptx hybrid confirms the successful loading of the drug. Upon the addition of Cur and Ptx drugs onto the GP cargo, significant peaks of Cur, Ptx as well as GP were observed in the FTIR spectra.

The successful functionalization of G with polymer was further validated using XRD (Supplementary Fig. S2). Native G has a broad band appearing at 2θ = 15–30°, which is due to van der Waal’s interaction between the carbon network on the graphene sheets^11^. The semicrystalline polymer, P, however, has two peaks at the same region, starting at ≈19–36° 18. Upon functionalization, these peaks appeared in the GP composite with an overlap of G and P bands at 15–19°. The peaks also appeared red-shifted compared to G and P alone.

The thermal stability of modified G was further analyzed by TGA (Supplementary Fig. S2). Up to 100 °C, a weight loss of 5% seen in G is due to the removal of adsorbed water molecules and the subsequent weight reduction from 200–400 °C is contributed by the bulk pyrolysis of carbon skeleton. Overall, G had a weight loss of approximately 87%. On the other hand, P suffered from 100% weight loss with obvious weight drop observed in the range of 270–400 °C, which is due to the decomposition of PEO and PPO copolymers^19^. However, upon functionalization with P, GP showed an enhanced thermal stability. Cur, which has a better heat resistance showed a weight loss of only 61% in the range of 516–822 °C. GP-Cur-Ptx showed an improved thermal stability due to the incorporation of Cur and Ptx. The thermal stability of the current synergistic system is comparable to the reported cyclodextrin modified Cur^20^.

**Figure 3:**
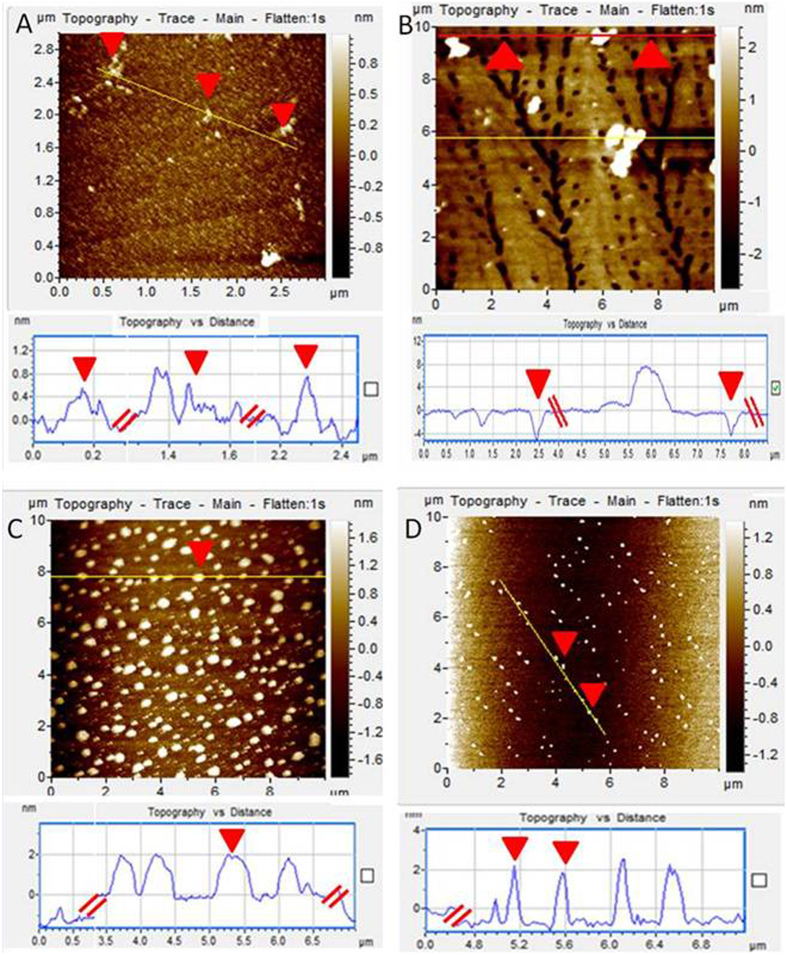
AFM images in tapping mode with the corresponding height profiles. (**A**) G, (**B**) GP, (**C**) GP-Cur and (**D**) GP-Cur-Ptx.

AFM in a tapping mode is a powerful tool to determine the material thickness and topography changes upon functionalization. G appeared with the thickness of ≈1 nm and upon modification with P, surface roughness was induced with the presence of a porous structure and an increased thickness of ≈4 nm (Fig. 3B). Accordingly, the height profile of GP-Cur is ≈2 nm and upon the introduction of Ptx has increased by ≈2–3 nm (Fig. 3D), confirming the non-covalent interaction of Ptx on the surface of GP. In addition, it is presumed that the molecules of Cur and Ptx which are smaller in sizes could have filled the pores that appear in GP.

The hydrophobic interaction between Cur and the cargo GP can be further ascertained in fluorescence emissions (Supplementary Fig. S3). Graphitic materials generally induce quenching upon interaction with aromatic or double bond conjugated materials, which is an imperative tool to confirm the loading of fluorescent materials or drugs. At an excitation wavelength of 420 nm, Cur exhibited a maximum fluorescence emission at ≈540 nm. However, in GP-Cur significant quenching of fluorescence was observed which is due to the strong pi-pi interaction between the conjugated system in Cur and the aromatic structures in GP. As a result, a photoinduced electron-transfer or fluorescence resonance energy transfer (FRET) takes place in the GP-Cur hybrid system that diminishes fluorescence^21^,^22^. In comparison, GP, Ptx and GP-Ptx did not demonstrate any fluorescence activity.

CV measurements of the GP-Cur-Ptx also revealed successful drug loading onto the GP cargo. Being an electrically conductive material, G has a higher current response with a broader CV area (Supplementary Fig. S3). Conversely, non–conductive materials such as P, Ptx and Cur have negligible CV responses. Upon functionalization with P, GP showed an enhanced electrical signal due to electron transfer with the double bond conjugated system that exists in the polymer. Similarly, GP-Cur, GP-Ptx and the synergistic system, GP-Cur-Ptx demonstrated a significantly higher CV response as compared to GP before drug loading. These enhanced current responses are attributed to the effective pi-pi stacking between the GP cargo and the loaded Cur and Ptx drugs.

**Figure 4:**
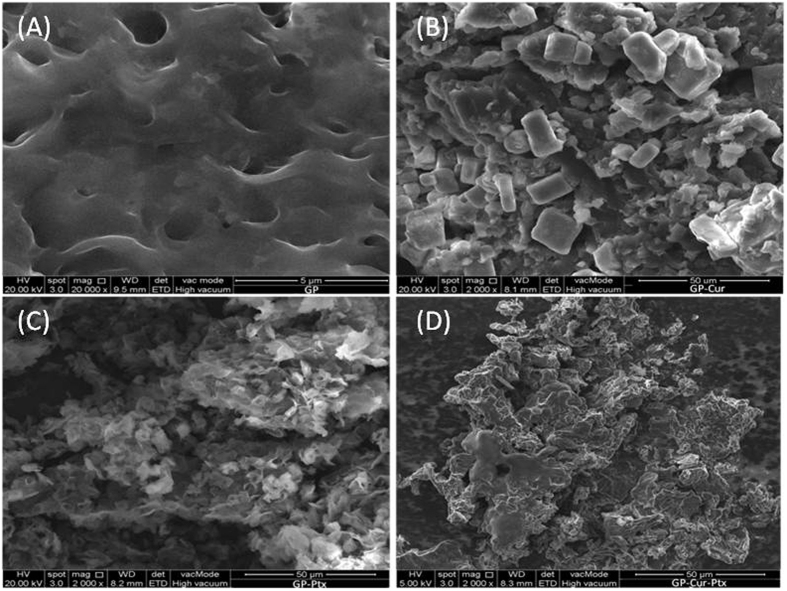
FESEM images of drug loaded GP. (**A**) GP, (**B**) GP-Cur, (**C**) GP-Ptx, and (**D**) GP-Cur-Ptx.

The surface morphology of GP-Cur-Ptx can be elucidated using FESEM (Fig. 4). After functionalization with P, the GP shows obvious surface roughness with microporous structure, which is consistent with the topographical observation under AFM. On the contrary, in the GP-Cur system, crystals of Cur in rectangular and cubic shapes appeared to decorate the surface of GP. Similarly, the appearance of granules in the GP-Ptx system confirms the successful loading of Ptx onto GP. In GP-Cur-Ptx, more homogenous and non-aggregated drug loading was observed.

The lateral and size distribution of aqueous dispersions of drug loaded GP were evaluated further using DLS (Supplementary Table S2). Although DLS is more suitable for the determination of spherical particles rather than the planar structures such as G, DLS is useful to indicate the size differences of G upon functionalization and to demonstrate whether uniformly-sized dispersion of materials is produced. Unmodified G has an average size of 144.7 nm and upon modification with P, the size of G slightly increased to 156.3 nm. In their unmodified forms, Cur (231.6 nm) and Ptx (148.0 nm) in ethanol/water have average sizes much larger than their hybrid form of GP-Cur (123.5 nm) and GP-Ptx (133.1 nm), respectively. As both are hydrophobic drugs, the aqueous dispersion is somehow limited, however upon loading onto GP, the solubility of these drugs was significantly improved and thus the lowered average sizes. Upon co-loading of Cur and Ptx drugs onto the GP cargo, the hydrodynamic average size increased to ≈140 nm. Using the current GP as a delivery vehicle generates substantially smaller particle sizes compared to the reported Cur loaded lipid-polymer nanoparticles with the sizes of 184 nm^23^.

### Drug loading of Cur onto GP cargo

The highest Cur loading observed was 2 mg with the loading efficiency of ≈97% and loading capacity of 678 wt.%. Higher loading of Cur on GP was achieved as compared to other recently reported delivery cargos^24^,^25^,^26^,^27^,^28^,^29^,^30^,^31^ (Supplementary Table S3). Even by utilizing GO, only 208 wt.% was obtained^24^ achieving only half of the loading capacity as compared to our current approach. As described, this remarkably higher drug loading is mainly due to the unperturbed surface present on GP contributing to the efficient pi-pi interaction with the Cur molecules. Ptx loading efficiency was determined as 98.00% ± 0.15.

### Comparison of activities of Cur and GP-Cur-Ptx based on DPPH assay

To ascertain that co-loading does not affect Cur’s bio-accessibility and hence its activity, DPPH studies were conducted on the synergistic system i.e. GP-Cur-Ptx and Cur for comparison. In addition to the presence of two types of drugs in GP-Cur-Ptx, the comparatively bulky polymer could also probably hinder the active sites of Cur, which in turn would show a reduction in the radical scavenging activity. Remarkably, based on the DPPH assay the GP-Cur-Ptx showed high antioxidant activity, which was relative to the free Cur (Supplementary Fig. S4). This finding confirms that there is no over-crowding on the surface of GP cargo and the PEO arms of the polymer are sufficiently spaced to allow unperturbed activity of Cur.

### Drug release profile of Cur and Ptx from GP-Cur-Ptx

A rapid release of drugs was observed at the first 8 h for both Ptx and Cur at pH 4 and 7.4 (Supplementary Fig. S5). Thereafter, a sustained drug release was recorded for both drugs with Cur being released much faster than Ptx. By 48 h, almost 50% of Cur was released in comparison to 25% of Ptx in the acidic pH. Overall, a higher drug release was observed at pH 4 compared to at physiological pH, which is advantageous for drug delivery as the cancer cells thrive in an acidic environment. The slow release also suggests the good stability of the GP-Cur-Ptx hybrid system.

### Antiproliferative effect of GP-Cur-Ptx on cancer cells

**Figure 5:**
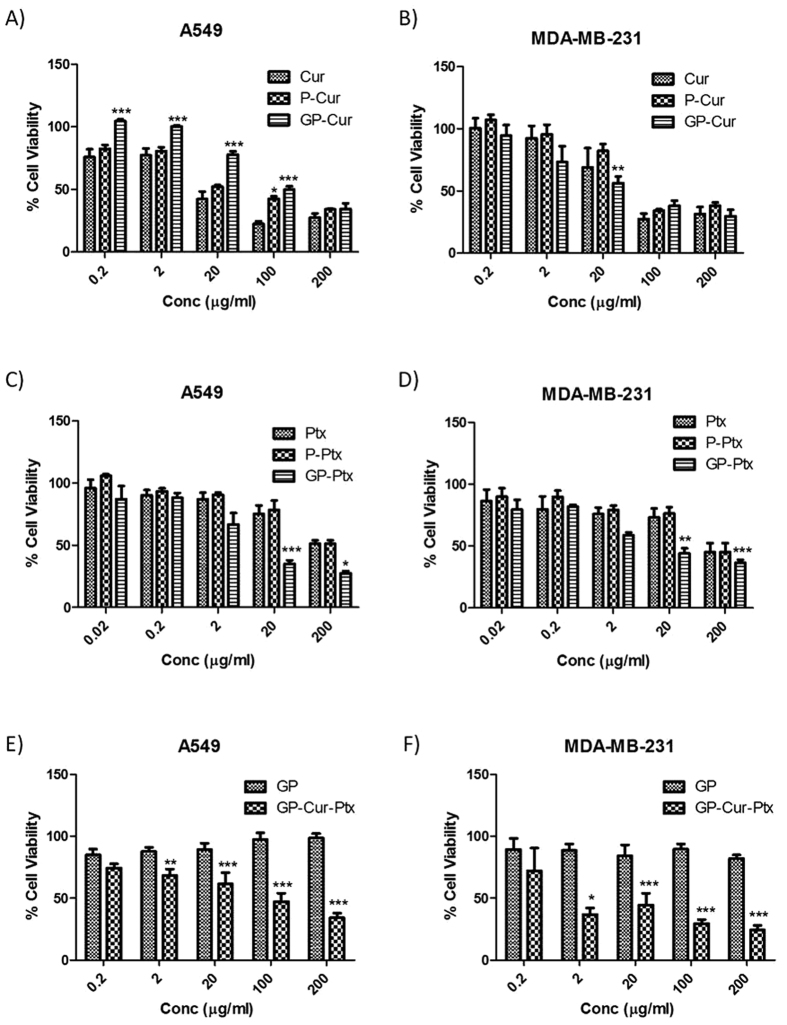
Representative histograms showing dose dependent growth inhibition. (**A**) A549 cells treated with Cur, P-Cur and GP-Cur, (**B**) MDA cells treated with Cur, P-Cur and GP-Cur, (**C**) A549 cells treated with Ptx, P-Ptx and GP-Ptx, (**D**) MDA cells treated with Ptx, P-Ptx and GP-Ptx, (**E**) A549 cells treated with GP and GP-Cur-Ptx and (**F**) MDA cells treated with GP and GP-Cur-Ptx. Following 48 h of treatment, IC_50_ was determined as described in Methods. Bars represent the mean ± SEM of triplicates from three independent experiments (n = 9). *, ** and *** indicate significant difference when compared between treatment groups, with P < 0.5, P < 0.01 and P < 0.001, respectively using two-way ANOVA with Bonferroni t-test.

A dose-dependent growth inhibition was evident at 0.2 μg/ml of GP-Cur in A549 and 20 μg/ml for MDA-MB-231 (MDA) cells (Fig. 5A,B). Significant growth inhibition was also observed at 20 ng/ml in cells treated with GP-Ptx, in contrast to 90% cell viability in cells treated with the same concentration of Ptx (Fig. 5C,D). Remarkably, an IC_50_ of 0.0077 μg/ml of GP-Ptx was observed against A549 cells representing almost 25-fold reduction in the required potent dose as compared to Ptx (Supplementary Table S4). The current GP-Ptx is highly efficacious as compared to the previously reported GO-PEG-Ptx, which needs at least 0.02 μg/ml to provide the same therapeutic effects in A549 cells^32^. Similarly, only 0.0148 μg/ml of GP-Ptx was needed to achieve IC_50_ in MDA cells, which is 8-fold lesser than using Ptx alone.

These findings raise the concern whether the markedly increased cellular toxicity of GP-Cur and GP-Ptx hybrid system was due to the toxicity from GP, instead of the loaded drugs i.e. Cur and Ptx. Cell viability studies showed that GP did not induce toxicity to A549 and MDA cells even at a higher concentration of 200 μg/ml (Fig. 5E,F). Morphological analysis of A549 and MDA cells treated with GP at 0.15 mg/ml (the maximum concentration used to prepare GP-Cur-Ptx) and at an even higher concentration of 0.30 mg/ml shows no signs of cell shrinkage or detachment, confirming its biocompatibility (Supplementary Fig. S6).

Since pluronic polymer has been suspected to be able to improve cellular uptake of drugs, Cur and Ptx loaded polymer (P-Cur and P-Ptx) was also taken as control (Fig. 5A–D). Based on the observation, the cytotoxicity of P-Cur and P-Ptx appears the same as Cur and Ptx, respectively. This concludes that in drug loaded polymer solutions, the drugs present as a mixture rather than a micelle formation with polymer. If the latter was formed, higher cytotoxic effect will be otherwise observed compared to Cur/Ptx.

With the introduction of 69.7 and 46.7 ng/ml of Ptx onto GP-Cur to the A549 and MDA cells, respectively, inhibition of cell growth was observed at 0.2 μg/ml (Fig. 5E,F). IC_50_ values showed that the GP-Cur-Ptx system was more potent towards MDA cells, requiring only 1.450 μg/ml of GP-Cur-Ptx compared to 13.24 μg/ml for A549 cells (Supplementary Table S5). The combination index (CI) was calculated based on the previously described method^33^, which showed synergistic inhibition of growth by GP-Cur-Ptx against the tested cells with slightly higher potency in MDA (0.43) as compared to A549 (0.54) cells (Supplementary Table S5).

In order to ascertain that the treatment is not toxic towards healthy cells, cytotoxicity test was also conducted against normal human lung fibroblast cells (MRC-5). GP, Cur and combined GP-Cur did not cause toxicity to non-cancerous MRC-5 cells, whereas Ptx and GP-Ptx induced toxicity at 0.4–0.5 μg/ml concentration (Supplementary Table S4). Cell morphology of MRC-5 treated with GP also shows no sign of toxicity (Supplementary Fig. S6).

To mimic the treatment for A549 and MDA cells, the same dose of GP-Cur-Ptx was tested against MRC-5 cells. Remarkably, at least 2 and 26-fold of higher concentration of GP-Cur-Ptx is required to induce toxicity to MRC-5 cells, as compared to the dose required in A549 and MDA cells, respectively (Supplementary Table S5). This clearly demonstrates that the combined treatment conferred less toxicity to non-cancerous MRC-5 cells as compared to the cancer cells, thus making the GP-Cur-Ptx system biocompatible to healthy cells.

### Determination of intracellular reactive oxygen species (ROS) generation in A549 and MDA cells

**Figure 6:**
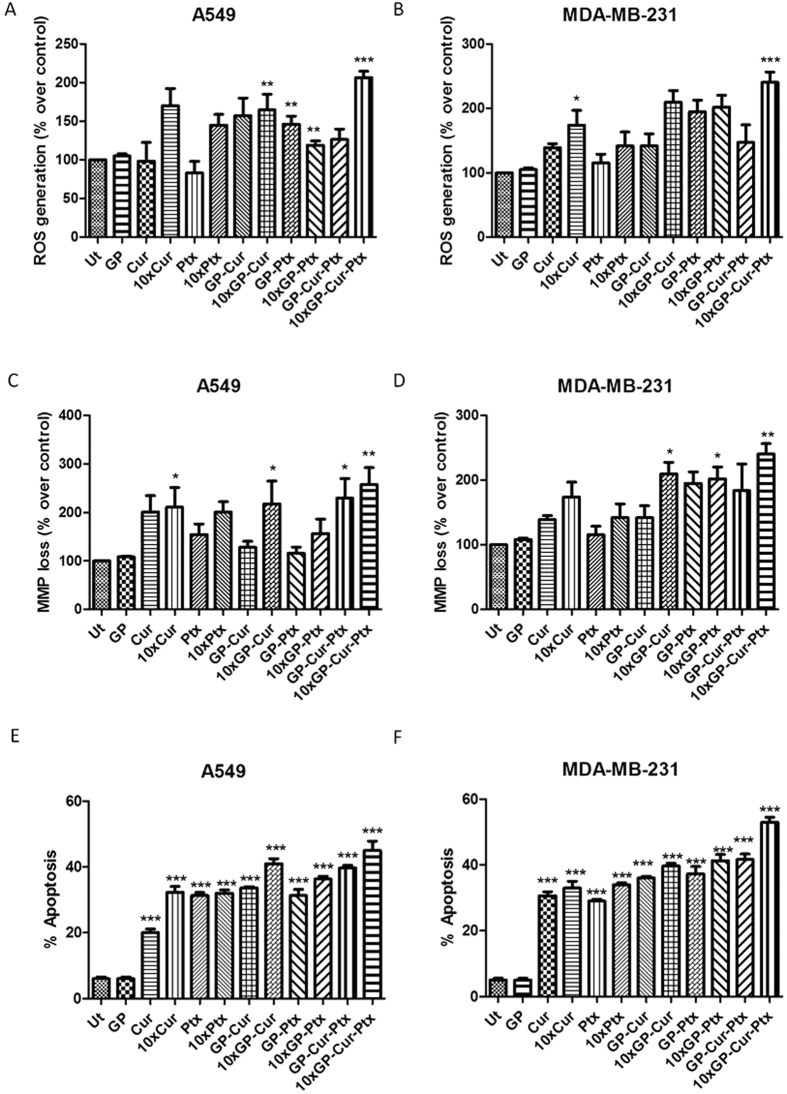
Percentages of ROS generation, loss of MMP and apoptosis. (**A**) Activity of ROS generation by the treatment groups on A549 and (**B**) MDA cells. (**C**) The loss of mitochondrial membrane potential (MMP) induced by treatment groups on A549 and (**D**) MDA cells. (**E**) Percentage of apoptosis induced by the treatment groups on A549 and (**F**) MDA cells. Plain media and GP served as untreated control (Ut) and negative control, respectively. Concentrations of the treatment groups are based on the respective IC_50_ and 10-fold IC_50_ values. Bars represent mean ± SEM, with n = 3. *, ** and *** indicate significant difference when treated groups were compared with un-treated with P < 0.5, P < 0.01 and P < 0.001, respectively using one-way ANOVA with Dunnett’s t-test.

The cytotoxicity of the treatment groups towards cancer cells was further confirmed with the determination of oxidative stress by fluorometric assay. In both cancerous cells, 10-fold GP-Cur-Ptx exuberantly elevated oxidative stress in the intracellular environment in a dose-dependent manner (Fig. 6A,B). In case of A549 cells, by comparing all the 10-fold IC_50_ treatment groups, 10-fold Cur demonstrated the second highest ROS elevation followed by 10-fold GP-Cur, which suggests that the presence of higher concentration of Cur has contributed to the amplified production of ROS. On the contrary, the drug carrier GP did not show any signs of ROS production (Fig. 6A,B), even though residual oxygen groups were present (based on the FTIR spectra), confirming its biocompatibility as observed in the cytotoxicity assay.

The findings of considerably higher ROS values as observed in the GP-Cur-Ptx corroborate well with the highest cell killing observed in the cytotoxicity assay, which indicates that the presence of Cur and Ptx have effectively induced toxicity to cancerous cells probably via a supplementary ROS-mediated mechanism.

**Figure 7:**
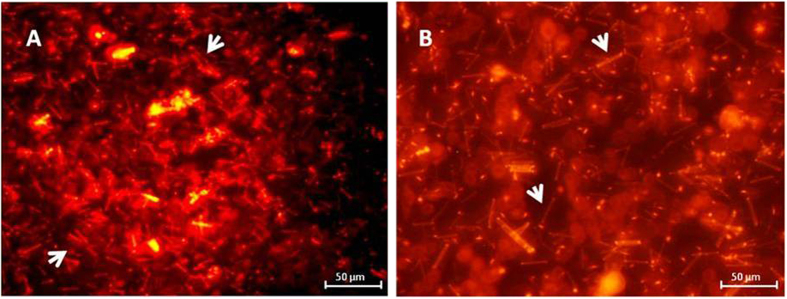
Representative images of ROS generation. (**A**) A549 and (**B**) MDA cells after treatment with GP-Cur-Ptx for 120 min. Upon incubation with cells, the generated ROS reacts with the fluorogenic component localized in the cytoplasm resulting in the generation of a red-fluorescent product, which in turn is proportional to the amount of ROS generated. Arrows indicate the rod-like structure as seen outside the cells, which are the GP cargo.

Figure 7 shows the ROS images of GP-Cur-Ptx on A549 and MDA cells. The brighter red fluorescence shows higher ROS generation and accumulation as compared to the unstained images of GP and untreated cells and lightly stained cells observed in GP-Cur and GP-Ptx (Supplementary Figs S7 and S8). The rod-like structure observed outside the cells shows the presence of GP which serves as a drug delivery vehicle and it corroborates with the hypothesis that only drugs were internalized by cells, leaving behind the GP cargo.

### Determination of mitochondrial membrane potential (MMP) loss in A549 and MDA cells

In both of the cancer cells tested, the highest dye permeability was observed in GP-Cur-Ptx (Fig. 6C,D), corroborating with the findings in ROS, which was also the highest. Loss of MMP was dose-dependent for GP-Cur-Ptx in A549 cells followed by 10-fold GP-Cur and 10-fold Cur. This indicates that the presence of Cur in these treatment groups has played a major role in disrupting the cells’ mitochondria. The current GP-Cur-Ptx combined treatments induced significantly higher depolarization of MMP as compared to the previously reported observations, a cocktail drug containing an antioxidant apigenin and paclitaxel^34^. As for MDA cells, GP-Cur-Ptx is as effective as 10-fold GP-Ptx in causing perturbation to the mitochondria, proving that the integrated treatment allows the usage of reduced dosages of Ptx to attain the same therapeutic effects.

### Determination of apoptosis in A549 and MDA cells

A549 and MDA cells treated with GP-Cur-Ptx showed the highest apoptosis (Fig. 6E,F), which is in agreement with the highest ROS generation and highest loss of MMP observed. The induction of apoptosis was also dose-dependent. The GP-Cur and GP-Ptx were more potent in inducing apoptosis as compared to the individual treatment of Cur and Ptx, even at 10-fold the concentration of IC_50_ values. To further comprehend the apoptotic trend, the cellular morphology of cells upon treatment was also investigated using a microscope.

**Figure 8:**
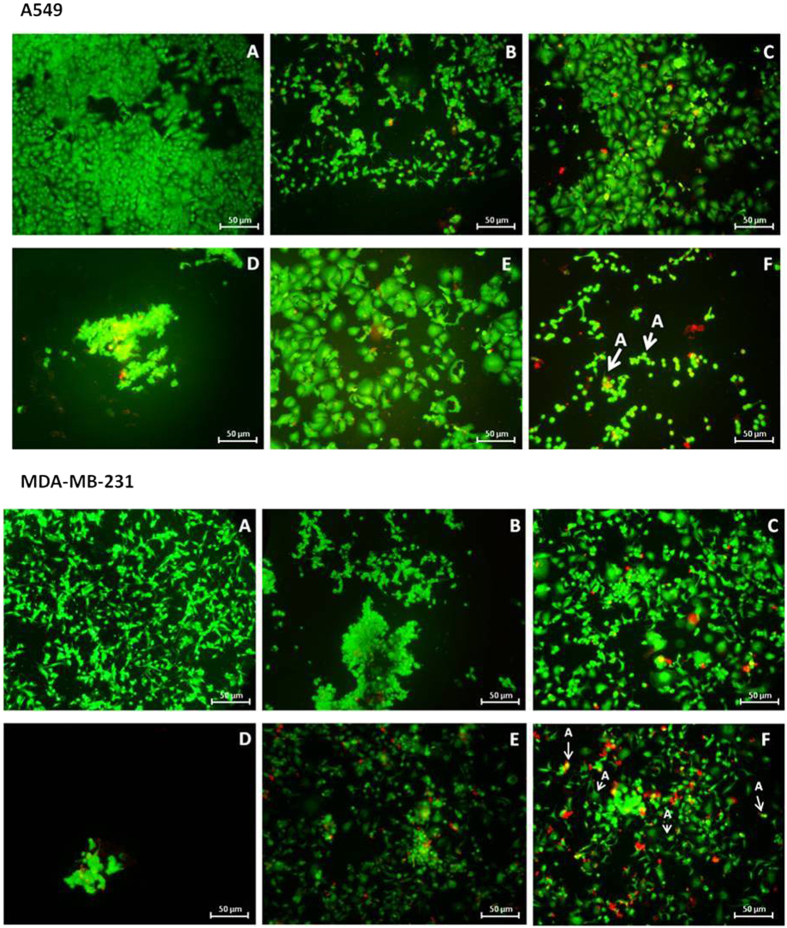
Merged images of A549 (upper panel) and MDA (lower panel) cells after staining with Annexin-Cy3 and 6-CFDA. (**A**) Untreated cells, and cells treated with: (**B**) Cur; (**C**) Ptx; (**D**) GP-Cur; (**E**) GP-Ptx and (**F**) GP-Cur-Ptx. Arrows labelled ‘A’ are representation of typical features of cells undergoing apoptosis. Cells that are green but show blebbing, cell-rupture or condensed and fragmented are taken as early stages of apoptosis, along with cells stained yellow in the merged images. Comparatively, cells stained red are regarded as necrotic.

For the untreated A549 and MDA cells, healthy cellular morphology was observed (Supplementary S9 and S10). Upon treatment, obvious separation from the neighbouring cells was seen with a higher separation constituting to higher apoptotic cell death. Both cancer cells that received treatment with Cur showed obvious signs of cell shrinkage (Fig. 8). A similar finding was also observed for the treatment with GP-Cur, along with the presence of yellowish-green in the merged images showing that the cells underwent early stages of apoptosis.

Treatment with Ptx induced the cells to undergo necrosis^35^ as more rampant red-staining was observed. Similarly, GP-Ptx showed the presence of necrosis with the treatment in MDA showing more rounded-up cells as compared to cells in A549. This is in accordance to the higher loss of MMP as seen in GP-Ptx for MDA cells. A549 and MDA cells treated with Ptx and GP-Ptx showed extensive plasma membrane blebbing, which is one of the late apoptotic characteristics. However, this was not found in the cells treated with Cur and GP-Cur.

Prominent features of cell apoptosis (shown by arrows) could be seen in the GP-Cur-Ptx treatments (Fig. 8F). Cellular shrinkage and pyknosis, were observed in both treated cancer cells. Additionally, blebs appeared more prominently in MDA cells, whereas karyorrhexis^36^ was seen more obviously in A549 cells. In conclusion, the GP-Cur-Ptx treatment on MDA cells caused late stages of apoptosis and necrosis, while A549 cells showed the characteristics of early stages of apoptosis. This finding could be translated to the higher level of ROS observed in MDA as compared to A549 cells. Cells treated with the drug carrier, GP, however does not show any signs of apoptosis, similar to the untreated control (Fig. S11).

## Discussion

Theoretically, the loading of drugs onto the GP cargo takes place due to pi-pi and hydrophobic interactions without any covalent functionalization. Hence, there is a concern whether the Cur were loaded onto the PPO groups of the polymer rather than on the surface of G, as the polymer also contains abundance of hydrophobic groups. The red shifts of GP-Cur as observed in UV-Vis and FTIR, the quenching of fluorescence, along with a higher electrical potential as noted in the CV analysis all confirmed that Cur is indeed loaded onto the surface of GP rather than the polymer. A similar finding was also observed in the GP-Ptx and GP-Cur-Ptx, corroborating an effective loading of the Cur and Ptx drugs onto the vacant surfaces of GP.

Another concern is whether the drug, Ptx was stacked on top of the Cur due to hydrophobic effects, instead of on the surface of G. It is postulated that this did not take place as the Ptx would have blocked Cur’s bio-accessibility and significantly lower the GP-Cur-Ptx’s DPPH activity, which was not observed here. This inference is also supported through the findings of CV analysis, where higher electrical response upon the addition of Ptx onto the GP-Cur hybrid system could be observed. In addition, there is also a concern of incomplete removal of polymer F127 which could contribute to micelle formation with Cur and Ptx. Despite dialysis and centrifugation of the GP system to remove unbound polymer, nevertheless, it is postulated that the presence of residual polymer could have not formed micelles with the drugs. This is evidenced by the observations in CV analysis. Upon loading of Cur/Ptx to the GP, a higher CV peak was observed compared to GP alone. The higher CV peak is mainly due to effective electron transfer between Cur/Ptx and the double bond conjugated system present in G. The possibility of a micelle formation with polymer and Cur/Ptx is eliminated as the higher CV curve would have not been observed in such a case. In the event of a micelle formation, the polymer which is nonconductive would have shown a low CV curve upon addition of Cur in the GP-Cur, GP-Ptx and GP-Cur-Ptx curves, respectively.

Moreover, pluronic polymers alone cannot be considered as an optimal delivery vehicle for hydrophobic drugs due to its own low solubility in aqueous media and short residence time in the physiological environment^37^. Thus, alterations such as polymeric micelles, covalent modifications or polymer thermogels based synthesis are necessary to encapsulate the hydrophobic drugs (Cur/Ptx). In this report, the drugs (Cur/Ptx) were loaded via simple mixing and bath sonication for 0.5 h, which is believed not sufficient to form micelle or covalent bond or thermogels with the residual pluronic polymer, if any. A similar finding was also previously reported^38^, whereby a non-covalent functionalized pluronic polymer-CNTs show no enhancement in cellular uptake by A549 cells compared to a covalent functionalized ones.

Thus, the enhanced cell toxicity effects as observed in GP-Cur and GP-Ptx hybrid systems could be due to the better solubilisation of Cur and Ptx upon loading onto the GP cargo system. Based on our previous report, the synthesized G has good dispersion and stability both in water and in the physiological solution which could have contributed to the better solubilisation of the loaded Cur and Ptx drugs^11^. In addition, the high surface area on G allows more reactive sites of the drugs available for the cytotoxicity reaction and possibly better cell internalization. The GP’s excellent biocompatibility compared to other reported work^39^ is suspected due to the synthesis route of GP, which is a green approach using mushroom extract as a reducing agent of GO.

The current GP-Cur-Ptx treatment is highly potent as compared to other reported delivery methods such as PLA-glycerol, where it induced toxicity to only 15% of MDA cells even at a higher concentration of 500 μg/ml^40^. Similarly, synergistic treatment of CKD712 and Ptx on MDA cells required 10 μg/ml and 100 ng/ml, respectively to reduce the cell proliferation by 50%^41^. Another attempt with natural anti-cancer agent luteolin co-delivered with Ptx required 15 μg/ml of luteolin and 40 ng/ml of Ptx to achieve IC_50_^42^. In these cases, higher drug concentrations were required to achieve the therapeutic effect as compared to the current GP-Cur-Ptx combination.

It was reported that Cur could either potentiate the accumulation of ROS or stimulate the generation of ROS by other anticancer drugs^43^. Although Cur possesses good ROS scavenging ability, in GP-Cur hybrid system Cur acts as a pro-oxidant by bringing cellular redox changes resulting in the accumulation of ROS, which consequently lead to cell death^44^. On the contrary, Ptx is reported to induce only a small amount of ROS due to the presence of a negative regulator of a mitochondrial ROS called UCP-2^45^. Hence, in the synergistic GP-Cur-Ptx system, Cur acts as an antioxidant which may have an adverse effect on the anticancer drug, Ptx which then acts on tumor cells by increasing the ROS level to induce cell death^46^. This is further supported by the drug release profile whereby Cur was released much faster than Ptx. The release of Cur in advance would allow effective chemosensitization of cancer cells which in turn would increase the therapeutic efficacy of Ptx^47^.

Based on the apoptosis studies, it can be deduced that Ptx and GP-Ptx induced cell necrosis in A549 and MDA cells, which is unfavourable as the cells are passive victims due to toxic process^48^. On the other hand, Cur and GP-Cur promotes cell apoptosis or programmed cell-death and with the introduction of Ptx in the synergistic treatment i.e. GP-Cur-Ptx, necrosis by Ptx was completely suppressed while promoting the signal-dependent cell suicide or apoptosis.

From the above findings, there is a consistent relationship between increased generation of ROS and increased loss of MMP that eventually lead to cell apoptosis and thus the inhibition of cell proliferation. The sequence of these events is however in need of further elucidation. Conceivably, it was reported that Cur and Ptx work synergistically in activating caspases, cleavage of Bid to tBid and mitochondrial release of cytochrome c, which consequently induce apoptosis by extrinsic and intrinsic pathways^49^.

In Summary, the chemical characterizations validate the successful loading of Cur and Ptx onto the GP cargo and a remarkably high drug loading was achieved. In addition, the loading of the drugs onto the GP has also increased its solubility.

The co-loaded system, GP-Cur-Ptx showed high potency against A549 and MDA cells, compared to treatments with single drugs. The same concentration tested on normal cells showed no toxicity, demonstrating that the GP-Cur-Ptx system is cell-specific cytotoxic. Upon further elucidation, the highest ROS production was observed in the GP-Cur-Ptx system, mainly owing to the presence of Cur. Based on cell morphological assessment, Ptx was found to induce late stages of apoptosis and necrosis in cells. However, in the combined treatment, cell necrosis by Ptx was subdued and the GP-Cur-Ptx majorly induced early stages of apoptosis. In addition, upon close examination, only the drugs were engulfed by cells, leaving behind the GP cargo. Further investigation is however necessitated in determining the actual reaction pathways that are activated by the GP-Cur-Ptx system, other than the commonly reported for Cur and Ptx.

## Methods

### Preparation of reduced graphene oxide (G)

G was synthesized based on our previously reported protocol^11^.

### Preparation of GP

PF-127 polymer (P) of 0.12 g was added to 20 ml of G (0.15 mg/ml), followed by bath sonication for 3 h. The reaction was then left under shaker overnight at 120 rpm. Subsequently, un-loaded P was removed by dialysis (MW cut-off = 14 kDa) against double-distilled water and left for a further 24 h. To further remove unbound polymer, ultracentrifugation at 10,000 rpm for 5 min was carried out. The final product, G functionalized with P will be named as GP hereafter.

### Cur loading studies

Cur in ethanol (2 ml) of various concentrations (0.1–2 mg/ml) was mixed with 2 ml of GP (0.15 mg/ml) and bath sonicated for 1 h, followed by mixing under shaker for another 1 h at 120 rpm. The resulting solution was centrifuged at 10,000 rpm for 15 min. The supernatant was then subjected to UV-Vis analysis, which represents the amount of Cur in excess. A Cur in ethanol calibration curve was initially obtained for quantification. The Cur loading efficiency is determined as


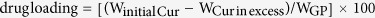



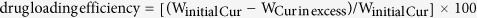


where, W_initial Cur_ is the initial weight of Cur added, W_Cur in excess_ is the weight of Cur in the supernatant and W_GP_ is the weight of GP. Samples were analyzed in triplicates.

### Preparation of Cur loaded GP (GP-Cur) and Ptx loaded GP (GP-Ptx)

Synthesis of GP-Cur was based on the maximum loading obtained from the above Cur loading studies. Cur (2 mg) was initially dissolved in DMSO/distilled water (1:1) solution. GP (0.15 mg/ml) solution was then introduced and the mixture was bath sonicated for 0.5 h before preparing various concentrations of GP-Cur with RPMI media as solvent. For comparison purposes, Ptx loaded onto GP (GP-Ptx) was also tested. For GP-Ptx, 1 ml of Ptx in DMSO (2 mg/ml) was added to 1 ml of GP (0.15 mg/ml), followed by bath sonication for 0.5 h and kept as aliquots at −20 °C. Cur and Ptx loaded onto polymer (P-Cur and P-Ptx) was also prepared as controls using the same protocol by replacing GP solution with polymer solution.

### Preparation of Cur and Ptx co-loaded onto GP (GP-Cur-Ptx)

IC_20_ of Ptx (determined from the cell proliferation assay) was added to the GP-Cur solution, followed by bath sonication for 0.5 h and dilutions as required in RPMI media. Briefly, IC_20_ of Ptx in A549 and MDA cells were determined as 69.7 and 46.7 ng/ml, respectively. Thus 69.7 and 46.7 ng/ml of Ptx was added to GP-Cur (0.2–200 μg/ml) to be tested against A549 and MDA cells, respectively. In order to determine the loading efficiency of Ptx on GP-Cur, 1 μg/ml of Ptx was bath sonicated with the prepared GP-Cur and the same protocol as Cur loading was followed with slight modifications. After centrifugation, the Ptx in the supernatant was extracted with dichloromethane and dried. The solid was then dissolved in acetonitrile/water (60:40) solution, filtered with 0.2 μm Millipore filter and then subjected to RP-HPLC (Agilent, USA) using a C18 column, 4.6 mm × 250 mm (Waters Corp., USA) equipped with a diode array detector. The mobile phase used was acetonitrile/water (60:40) with a flow rate of 1 ml/min. Amount of Ptx was determined via UV/Vis at 250 nm. A Ptx calibration curve was initially obtained for quantification. Drug loading efficiency was determined as mentioned previously. Samples were analyzed in triplicates.

### Determination of DPPH radical scavenging activity

The activity of Cur in the GP-Cur-Ptx system was monitored based on a previously reported method with slight modifications^50^. Briefly, concentration range of 1–200 μg/ml of Cur and GP-Cur-Ptx were prepared in ethanol, followed by 30 min of incubation with 0.1 mM DPPH in the ratio of 1:3. Triplicate experiments were performed with absorbance recorded at 517 nm and the obtained results were expressed as percentage of spectrophotometric DPPH quenching.

### Drug release studies

GP-Cur-Ptx (3 ml) was enclosed in a dialysis membrane with a molecular weight cut-off of 14 kDa and transferred to 50 ml of PBS buffer (pH 4 and 7.4) each, containing 0.2% Tween 80 to maintain sink conditions. It was then placed in a water bath at 37 °C under gentle agitation, following which at predetermined intervals; the release medium was removed and replaced with fresh buffer. The amount of Ptx and Cur released was determined by extracting the release medium with dichloromethane and subjecting to HPLC analysis as previously mentioned. The absorbance wavelength for Ptx and Cur was observed at 250 and 420 nm, respectively. All the samples were analyzed in triplicates.

### Cell culture

MRC-5 (human normal lung fibroblast), MDA-MB-231 (MDA, human breast adenocarcinoma) and A549 (human lung adenocarcinoma) cells were obtained from ATCC (Manassas, USA) and cultured in RPMI media supplemented with 10% FBS and 1% penicillin/streptomycin. All cell lines were maintained at 37 °C in a humidified 5% CO_2_ atmosphere.

### Determination of cell proliferation using neutral red uptake assay

A total of 5 × 10^3^ of MRC-5, MDA and A549 cells were cultured in 96-well plates, followed by incubation overnight for cell attachment. The cells were then exposed to 200 μL of various concentrations of the treatment groups (GP-Cur, GP-Ptx, GP-Cur-Ptx) and further incubated for 48 h. For comparison purposes, unmodified Cur and Ptx were also tested. Cells grown in media without any treatment served as negative control. Following 48 h of incubation, the cell viability was determined using the neutral red uptake assay according to the previously described protocol^51^. The IC_50_ values were determined using the non-linear regression curve fit of the Graphpad Prism5 software. The morphology changes of cells after treatment with GP (0.15 and 0.30 mg/ml) for 48 h were taken with contrast-phase microscopy, Nikon Az100 (Japan).

### Determination of intracellular ROS generation

The generation of intracellular ROS was determined using MAK 142 fluorometric kit (Sigma Aldrich, USA). A549 and MDA cells were seeded at a density of 5 × 10^4^ cells in black 96-well plates with clear bottom. In a preliminary study, the ROS generation peaked at 120 min, thus taken as the incubation time. Cells were treated with IC_50_ and 10-fold IC_50_ doses of respective treatments (Cur, GP-Cur, Ptx, GP-Ptx, GP-Cur-Ptx) and GP (0.15 mg/ml) as well as plain media (untreated) cells served as negative controls. Following incubation for 120 min, the ROS generated were determined according to the manufacturer’s protocol. Subsequently, the fluorescence intensity was quantified using a fluorescence Varioskan flash microplate reader (Thermo scientific, USA) with excitation and emission wavelengths fixed at 650 and 765 nm, respectively. ROS generation was determined as the percentage of ROS compared to control. For fluorescence microscopy imaging, A549 and MDA cells were seeded in 4-well chamber slides (SPL Life Sciences, South Korea). ROS reagents of 125 μL were used and images were captured using Nikon Az100 fluorescence microscope (Nikon, Japan).

### Determination of MMP depletion

A total of 5 × 10^4^ A549 and MDA cells were seeded in black 96-well plates with clear bottom and were allowed to attach overnight. The cells were then exposed to 200 μL of treatment groups with the concentration of IC_50_, 10-fold IC_50_ and plain media (untreated control) and GP (0.15 mg/ml) to monitor the effect of increased dose. After 48 h of incubation, MMP in terms of permeability of JC-10 dye was quantified by using MAK159 MMP kit according to the manufacturer’s protocol (Sigma Aldrich, USA). JC-10 forms reversible red-fluorescent aggregates in healthy cells with polarized mitochondrial membrane, however, upon the collapse of MMP, JC-10 returns to its monomeric green-fluorescent form^52^. MMP loss for each treatment group is calculated as a ratio of green (λ_ex_ = 490/λ_em_ = 525 nm) to red (λ_ex_ = 540/λ_em_ = 590 nm) fluorescence and was taken as a percentage over control.

### Determination of apoptosis using Annexin-V assay

A549 and MDA cells were seeded at a density of 5 × 10^4^ cells in 4-well chamber slides and treated with IC_50_ and 10-fold IC_50_ doses in comparison with an untreated control and GP (0.15 mg/ml) for 24 h. Thereafter, apoptosis was detected by using APOAC Annexin V-Cy3 apoptosis detection kit according to the manufacturer’s protocol (Sigma Aldrich, USA) with slight modifications. Briefly, treated cells were washed twice with PBS (100 μL) and three times with 100 μL of binding buffer. Subsequently, 100 μl of double-label staining solution containing Annexin Cy-3 and 6-Carboxyfluorescein diacetate (6-CFDA) was then added and incubated for 10 min at RT to quantify the living cells from apoptotic and necrotic cells. The staining solution was then removed and the cells were washed five times with 50 μL of binding buffer. Finally, 50 μL of binding buffer was placed in each well and covered with cover slips and images were captured using Nikon Az100 fluorescence microscope fitted with TRITC filter (Nikon, Japan). Annexin Cy-3 stains red on necrotic cells, by binding to phosphatidylserine, which is present outside the plasma membrane of cells undergoing apoptosis. Upon entering living cells, the non-fluorescent 6-CFDA is hydrolyzed by esters producing a green-fluorescent product. Cells in the early stages of apoptosis, however, stained yellowish-green^53^.

## Additional Information

**How to cite this article**: Muthoosamy, K. *et al*. Exceedingly Higher co-loading of Curcumin and Paclitaxel onto Polymer-functionalized Reduced Graphene Oxide for Highly Potent Synergistic Anticancer Treatment. *Sci. Rep.* **6**, 32808; doi: 10.1038/srep32808 (2016).

## Supplementary Material

Supplementary Information

## Figures and Tables

**Figure 1:**
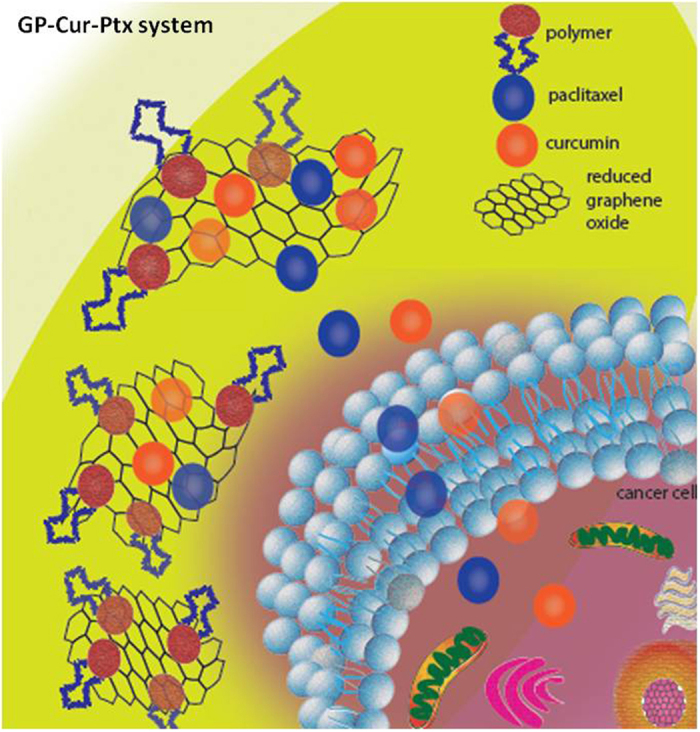
Representative image of drug delivery. Cur and Ptx delivery into cancer cells using a GP cargo.
