# Supplementary material for: Exceedingly Higher co-loading of Curcumin and Paclitaxel onto Polymer-functionalized Reduced Graphene Oxide for Highly Potent Synergistic Anticancer Treatment
Source: Sci Rep. 2016 Sep 6;6:32808. doi: 10.1038/srep32808 (PMC5011726; doi:10.1038/srep32808)
Supplement: Supplementary Information [file srep32808-s1.pdf]

Supporting Information for:

Exceedingly Higher co-loading of Curcumin and Paclitaxel onto Polymer-functionalized Reduced Graphene Oxide for Highly Potent Synergistic Anticancer Treatment

Kasturi Muthoosamy,[1] *Ibrahim Babangida Abubakar,*[2] *Renu Geetha Bai,*[1] *Hwei-San Loh* [2,3] *and Sivakumar Manickam* [1]

[1]Centre for Nanotechnology and Advanced Materials (CENTAM), Faculty of Engineering, University of Nottingham Malaysia Campus (UNMC), 43500 Semenyih, Selangor, Malaysia.

[2]School of Biosciences, Faculty of Science, UNMC, 43500 Semenyih, Selangor, Malaysia.

[3]Biotechnology Research Centre, UNMC, 43500 Semenyih, Selangor, Malaysia.

*Correspondence should be addressed to:

Sivakumar Manickam,
Email: Sivakumar.Manickam@nottingham.edu.my
Tel: +60389248156, Fax: +60389248017

Materials

Reduced graphene oxide (G) was synthesized based on our previously reported protocol.[23] Ultrapure deionized water was obtained from Milli-Q Plus system (EMD Millipore, Billerica, MA, USA). Phosphate buffered saline (PBS) (without Ca and Mg), dimethylsulfoxide (DMSO), RPMI 1640 and Trypsin-EDTA of tissue culture grade were obtained from Nacalai Tesque (Japan). Fetal bovine serum (FBS) was obtained from JR Scientific (USA). Paclitaxel (Ptx), curcumin (Cur), pluronic^{R} F-127 (P), 1,1-diphenyl-2-picrylhydrazyl (DPPH), Tween 80, dichloromethane and acetonitrile were obtained from Sigma Aldrich (USA). All chemicals used were of biological grade and with the highest purity

Characterizations

The functionalization of G with P and the subsequent loading of drugs onto the cargo were monitored by ultraviolet-visible (UV-Vis) spectroscopy using a Lambda 35 Spectrophotometer (Perkin Elmer, USA). All the materials were dissolved in water/ethanol (1:1) mixture before subjecting to UV-Vis measurements. Ptx, however, was initially dissolved in DMSO. The crystallographic state of G, P and GP was investigated using an X'Pert Pro X-ray powder diffractometer (XRD) (PANalytical, Netherlands). The XRD was operated at 45 kV with the current of 35 mA using Cu/kα radition (λ=1.54060 Å). Fourier transform infrared (FTIR) spectra were recorder on a Perkin Elmer, Spectrum RX1 in the frequency range of 4,000-400 cm^{-1}. The thickness of the material was examined using an atomic force microscopy (AFM) 5500 System (Agilent Technologies, USA) in a tapping mode. The samples were spin-coated on a freshly cleaved mica substrate before subjecting to AFM analysis. The freeze-dried samples were subjected to morphology analysis using field emission gun electron microscopy (FESEM) on a Quanta SEM 400 instrument (FEI, USA). The difference in

electrochemical potential upon modification of G was determined by cyclic voltammetry (CV) measurements with a Princeton Verstastat electrochemical station. Ag/AgCI was used as reference electrode, platinum wire as counter electrode and bare and modified glassy carbon electrode (GCE) as the working electrode. Potential range was set at -0.1 V to 0.6 V with a scan rate of 50 mV/s and PBS was used as the electrolyte. Results were collected after 10 CV cycles. The relative hydrodynamic diameter of all the materials was identified from the dynamic light scattering (DLS) technique using a Zetasizer Nano ZS (Malvern Instruments, UK). Thermogravimetric analysis (TGA) was performed using a TGA/differential scanning calorimetry 1, Stare System (Mettler Toledo Inc., USA). Samples were analysed under nitrogen flow (50 mL/min) and were heated from room temperature (RT) to 1000°C at 5°C/min.

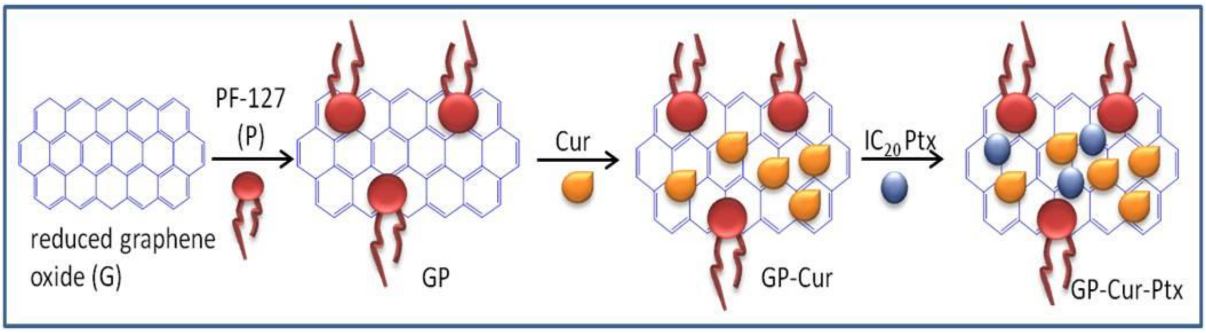

Figure S1: Polymer functionalization of G and followed by drug loading, forming a GP-Cur-Ptx cargo.

Table S1: Assignment of FTIR spectra of G, P, Cur and Ptx as presented in Figure 1B.

Materials	IR absorption bands (cm^{-1})	Peak assignment
G	1593	C=C stretching
	1383	CH bending
P	3480	OH stretching
	2954	CH stretching from CH_3
	2870	CH stretching from CH_2
	1476	CH bending
	1352	CH_2 wagging, C-C stretch
	1276, 1248	CH_2 twisting
	1222-978	C-O stretching from C-O-C
	1100	CO stretching from ROH
	962	CH_2 rocking, C-O-C stretching
	838	CH_2 rocking
Cur	3509	OH stretching band of phenol
	3024	CH stretching band
	2982	Asymmetric CH stretch of CH_3
	2946	CH stretch of OCH_3
	1624	C=C, C=O stretching mode
	1600	C=C stretching vibration of aromatic ring
	1505	C=O stretching mode, CCC and CC=O in-plane bending
	1452, 1429, 1370	CH in-plane bending vibrations, skeletal CCC, aromatic CCC and CCH modes
	1275	CO enol stretching
	1240-980	COC stretching, out of-plane bending of CH_3, in-plane bending of aromatic CCH bands
	959	CH trans vibration in benzoate
	856-720	$RCH=CH_2$ bending vibrations
Ptx	3476	NH stretching, OH stretching
	2976-2833	CH_2 symmetric and asymmetric stretching
	1728	C=O stretching from RCOOR
	1666	C-C stretching, N-H bending
	1383	CH_3 bending
	1298	CO stretching from RCOOR
	1237	CN stretching
	1170-1070	CO stretching
	1070, 967, 719	CH out of plane deformation
	719	C-C=O deformation

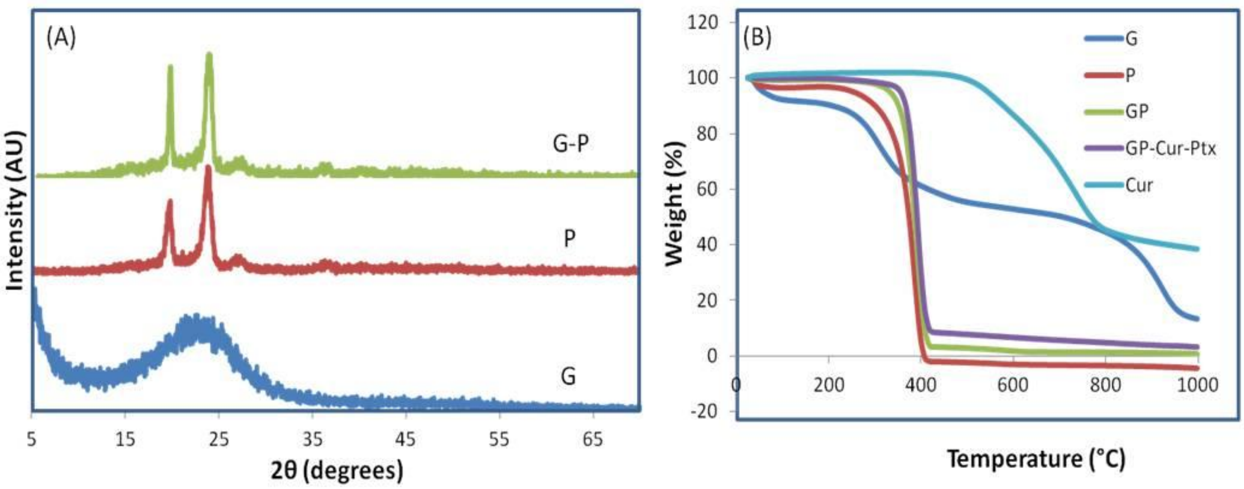

Figure S2: (A) XRD patters of G, P and GP (B) TGA curves of the functionalized GP and drug loaded GP.

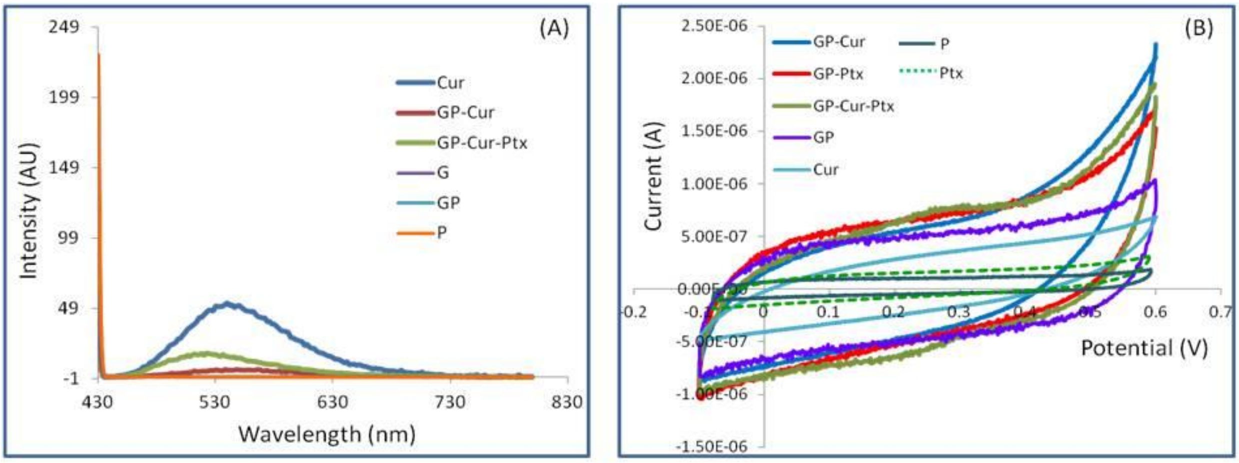

Figure S3: (A) Fluorescence spectra and (B) CV analysis of Cur and Ptx loaded onto GP.

Table S2: The average hydrodynamic diameter of G and modified G and the respective polydispersity index (PDI) after average of 9 separate analyses.

**Sample**	**Average size (nm)**	**PDI**
G	144.7 ± 0.3	0.140 ± 0.030
GP	156.3 ± 0.3	0.111 ± 0.130
Cur	231.6 ± 0.5	0.019 ± 0.007
Ptx	148.0 ± 0.7	0.038 ± 0.013
GP-Cur	123.5 ± 0.5	0.245 ± 0.050
GP-Ptx	133.1 ± 0.9	0.078 ± 0.076
GP-Cur-Ptx	139.9 ± 0.4	0.063 ± 0.089

Table S3. Recent reports on the types of vehicles used for the delivery of Cur.

**Functionalization**	**Encapsulation Efficiency/Loading Efficiency (%)**	**Ref.**
Protein-pectin	86	[25]
Cremophor	94	[26]
Catechin	88	[27]
Gelatin	48	[28]
Poly(ethylene glycol)-poly(lactic acid) (PEG-PLA)	8.6	[29]
PLGA-castor oil	92	[30]
MPEG-P(CL-co-TMC)	96	[31]
GO (graphene oxide)	42	[24]
GP (polymer-functionalized reduced graphene oxide)	96.56 ± 0.07	Present report

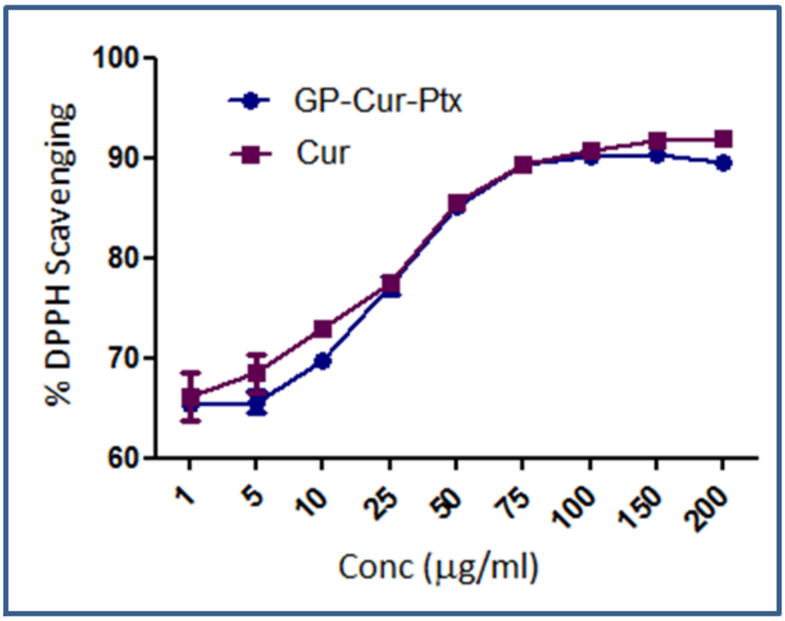

Figure S4: DPPH activity assay of unmodified Cur and GP-Cur-Ptx. Data presented are mean ± SD of triplicates (n=3).

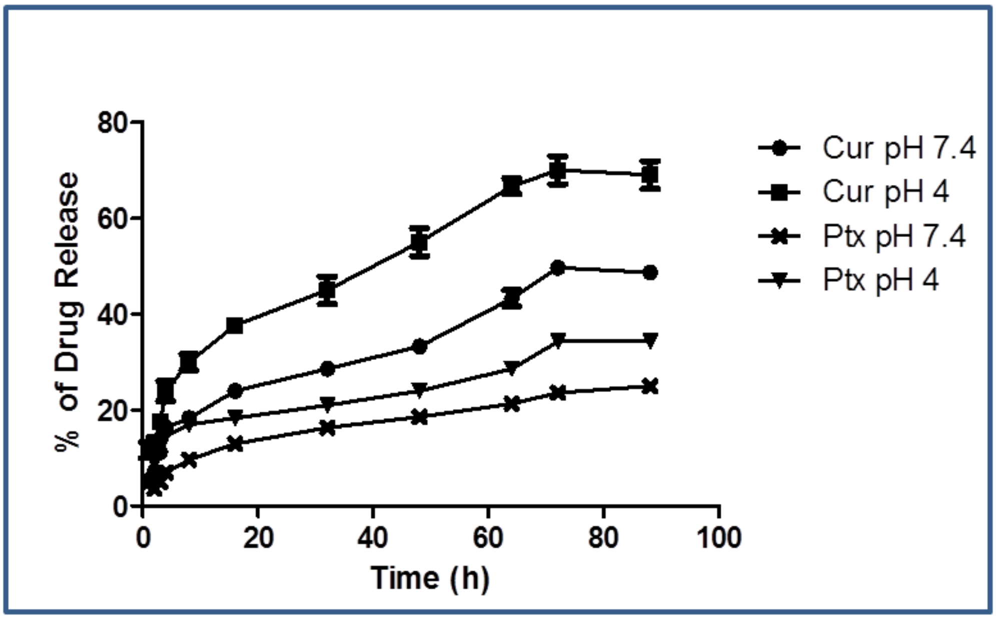

Figure S5: Release profile of Cur and Ptx from the GP-Cur-Ptx system in PBS buffer at pH 4 and 7.4, monitored for 90 h. Data presented are mean ± SD of triplicates (n=3).

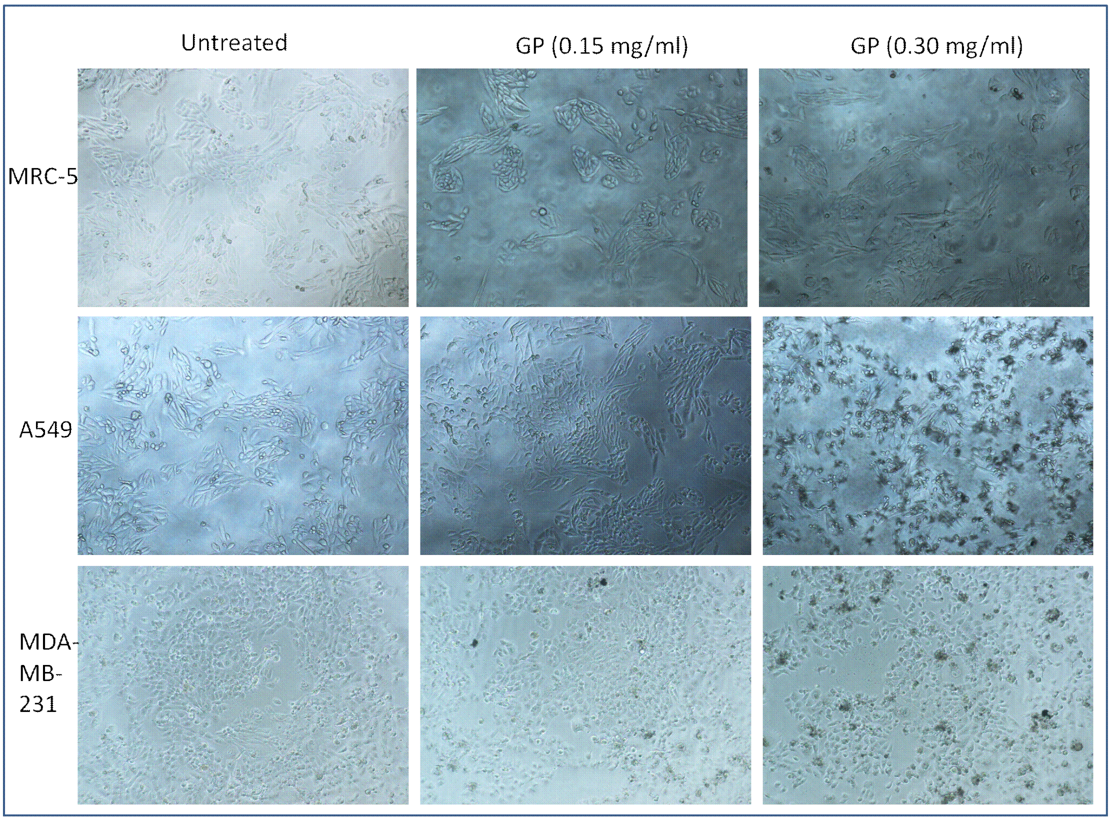

Figure S6: Morphology images of untreated cells and cells treated with the drug carrier, GP at 0.15 mg/ml for MRC-5, A549 and MDA-MB-231 cells. No changes in morphology were observed even at a higher concentration of 0.30 mg/ml, except darkening of the cell solution due to the presence of black coloured GP.

Table S4. The 50% inhibitory concentration (IC_{50}) values of Cur and Ptx; Cur and Ptx loaded onto GP (GP-Cur and GP-Ptx) and Cur and Ptx co-loaded onto GP (GP-Cur-Ptx), tested against A549, MDA and MRC-5 cells.

**Treatment (µg/ml)**	**A549**	**MDA**	**MRC-5**
Cur	17.83 ± 1.4	49.42 ± 1.3	> 200
GP-Cur	96.13 ± 1.1	42.98 ± 1.4	> 200
Ptx	0.1743 ± 0.0013	0.1174 ± 0.0016	0.5237 ± 0.0014
GP-Ptx	0.0077 ± 0.0015	0.0148 ± 0.0016	0.4041 ± 0.0012
GP	> 200	> 200	> 200

Table S5. Combination index (CI) analysis of GP-Cur-Ptx against A549 and MDA cells and the effect of these respective doses on MRC-5 cells, in terms of IC_{50} values.

No.	Cells	IC_{20} doses of Ptx (ng/ml)	Doses of GP-Cur which induced 50% growth inhibition in combination with IC_{20} dose of Ptx (μg/ml)	CI
1	A549	69.7	13.24 ± 1.8	0.54
2	MDA	46.7	1.450 ± 1.9	0.43
3	MRC-5	69.7	25.71 ± 1.2	-
4	MRC-5	46.7	37.50 ± 1.2	-

Note: Pharmacological interaction deduced the CI values of >1 as antagonistic, =1 as addictive and <1 as synergistic.

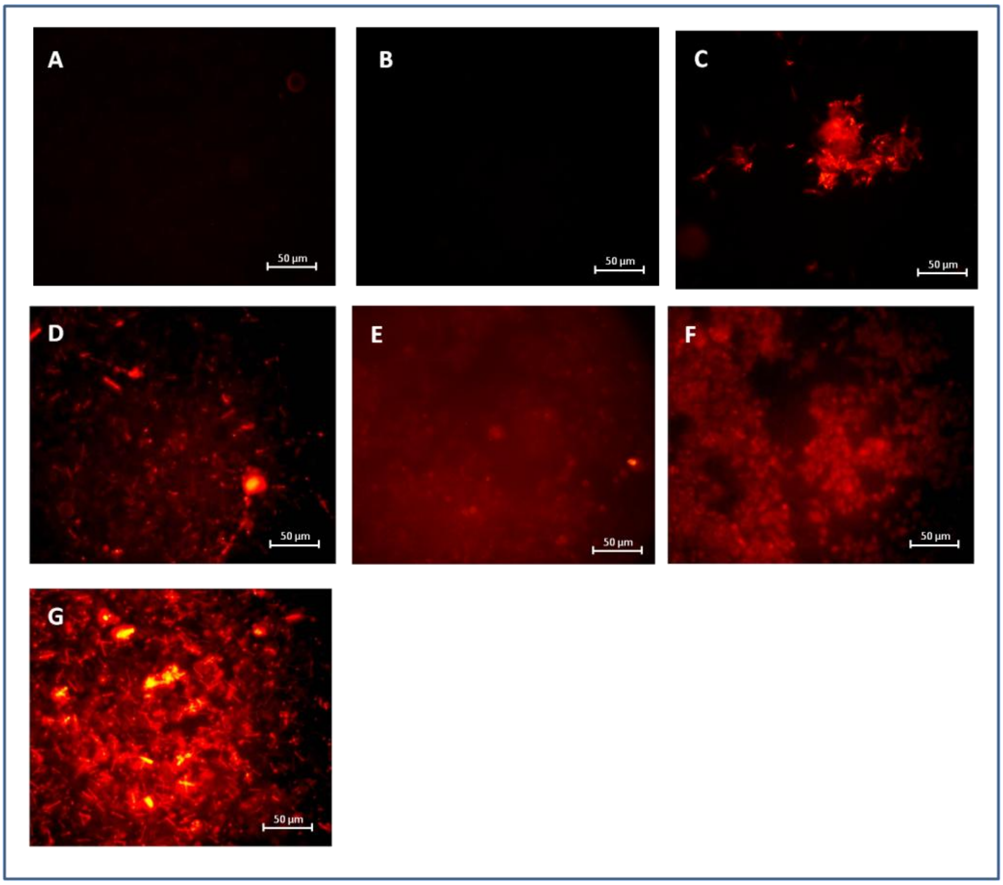

Figure S7: ROS generation images of A549 cells after 120 min treatment with (A) media alone (untreated), (B) GP alone, (C) Cur, (D) GP-Cur, (E) Ptx, (F) GP-Ptx and (G) GP-Cur-Ptx.

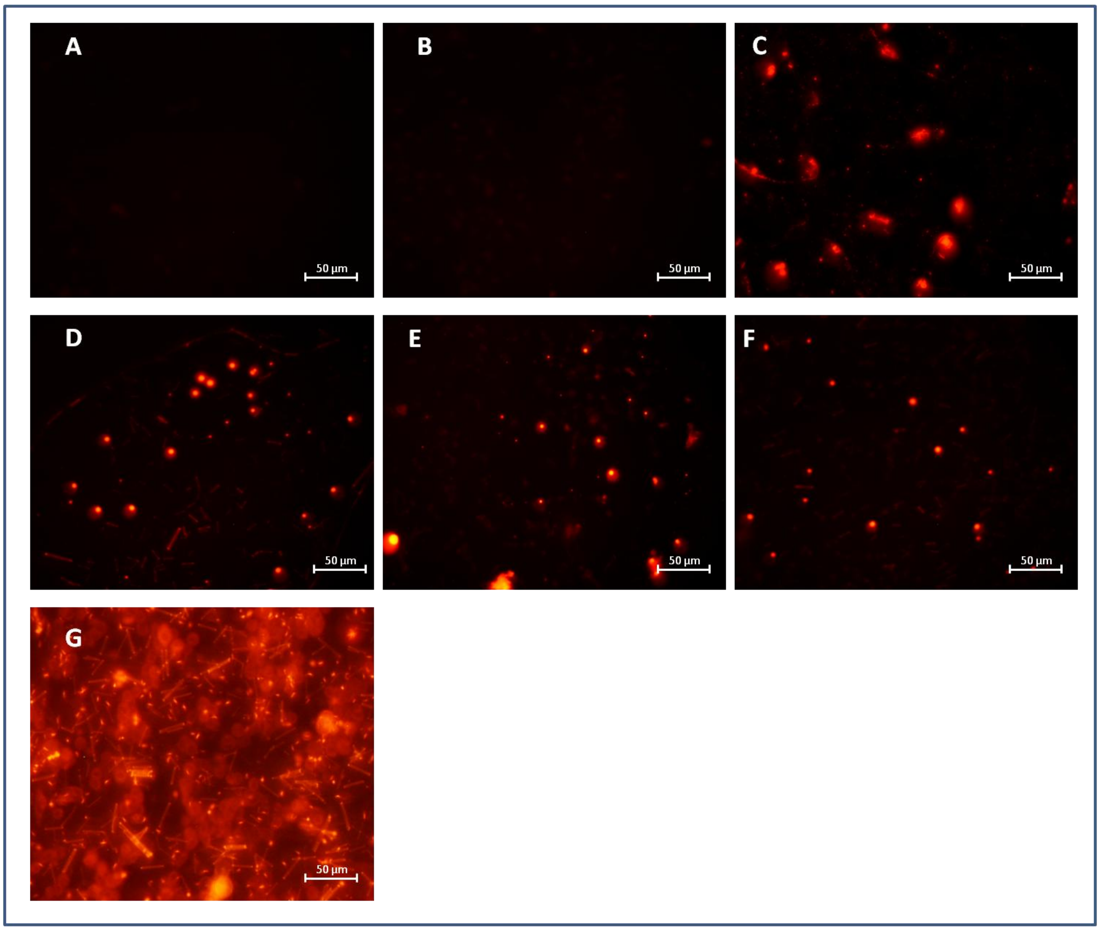

Figure S8: ROS generation images of MDA-MB-231 cells after 120 min treatment with (A) media alone (untreated), (B) GP alone, (C) Cur, (D) GP-Cur, (E) Ptx, (F) GP-Ptx and (G) GP-Cur-Ptx.

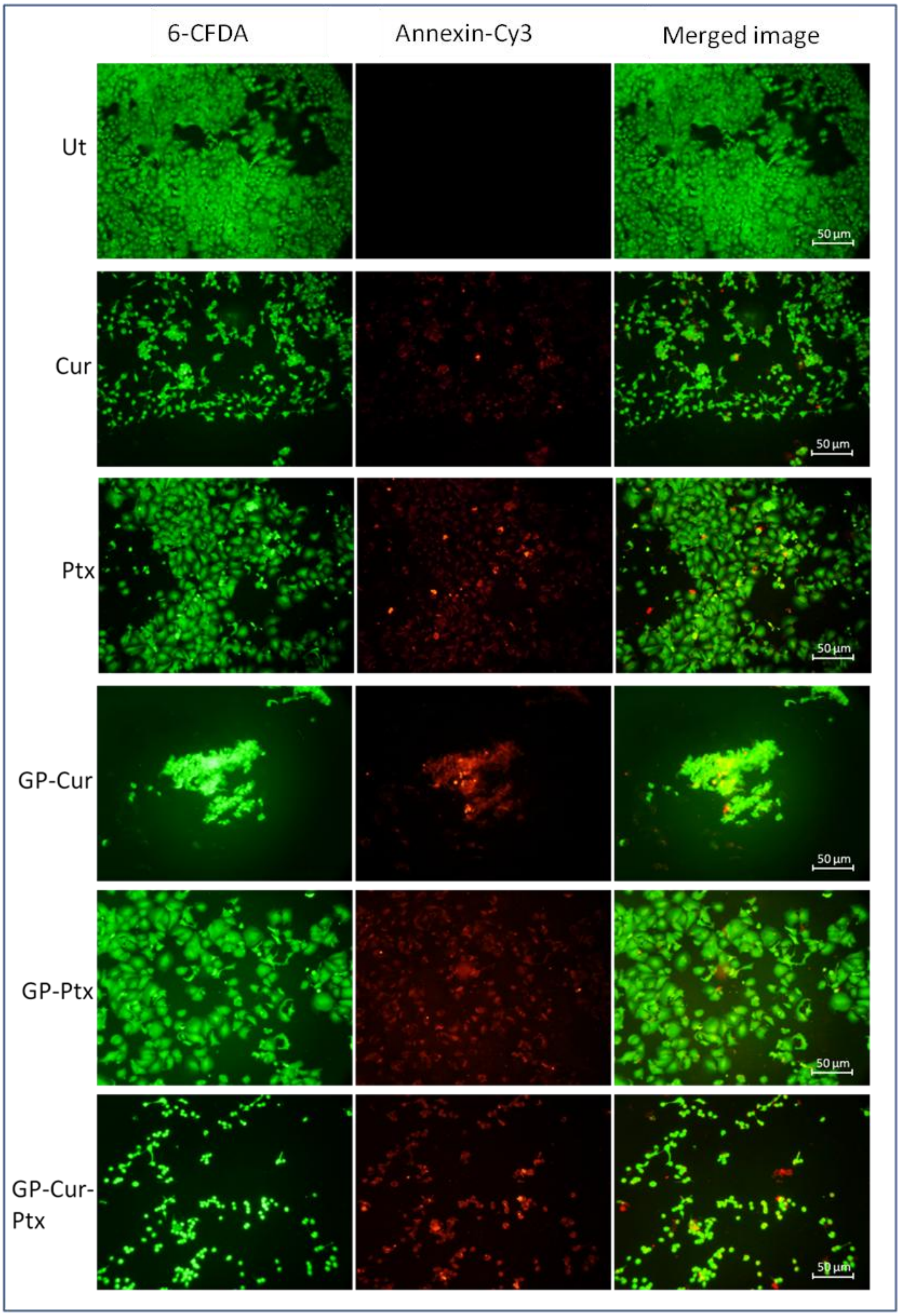

Figure S9: Images of untreated (Ut) A549 cells and A549 cells treated with Cur, Ptx, GP-Cur, GP-Ptx and GP-Cur-Ptx. The green fluorescent (left panel) represents the non-apoptotic cells. The red fluorescent (middle panel) represents necrotic cells and the yellowish-green fluorescent seen in the overlay images (right panel) represents early stages of apoptosis.

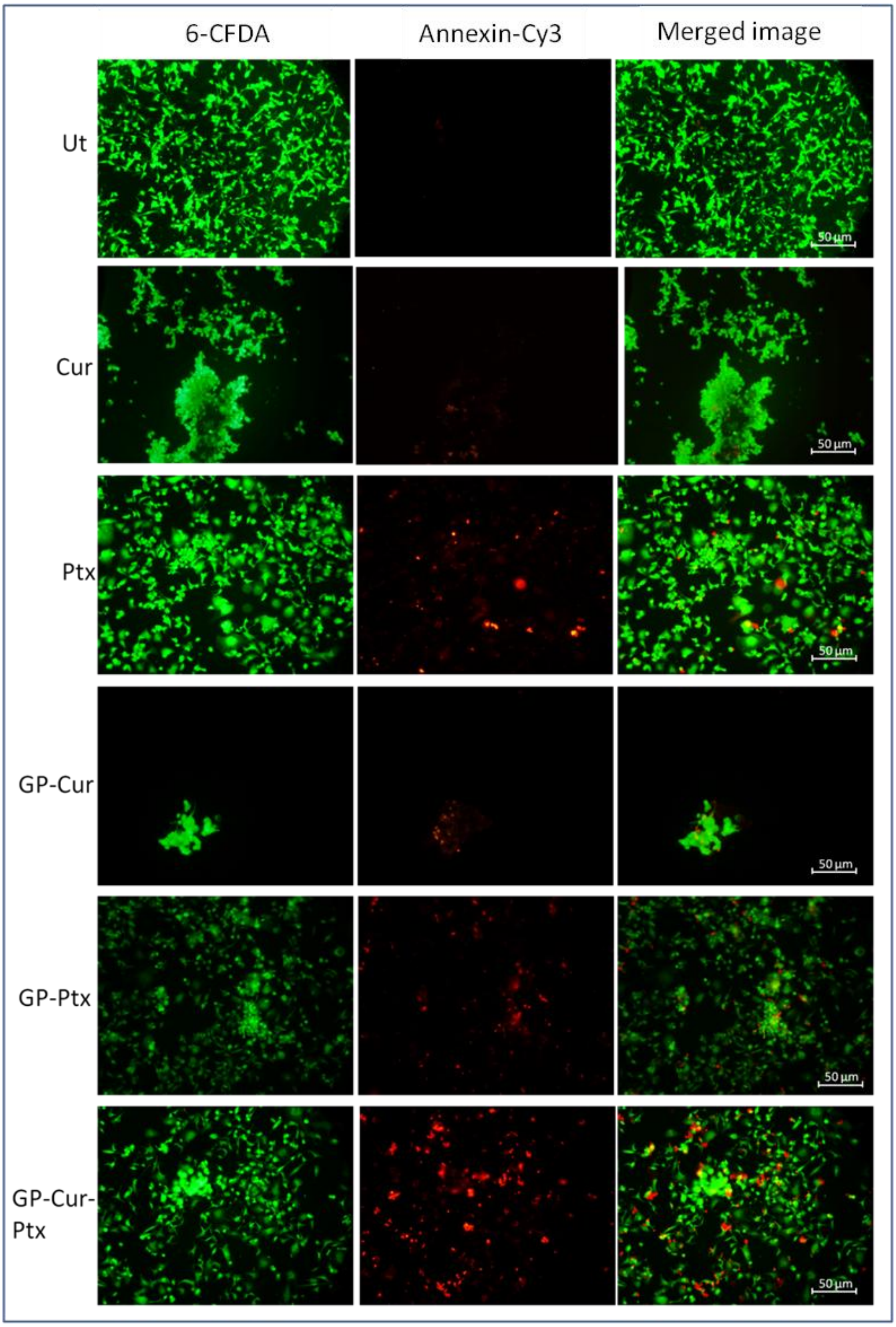

Figure S10: Images of untreated (Ut) MDA-MB-231 cells and MDA-MB-231 cells treated with Cur, Ptx, GP-Cur, GP-Ptx and GP-Cur-Ptx. The green fluorescent (left panel) represents the non-apoptotic cells. The red fluorescent (middle panel) represents necrotic cells and the yellowish-green fluorescent seen in the overlay images (right panel) represents early stages of apoptosis.

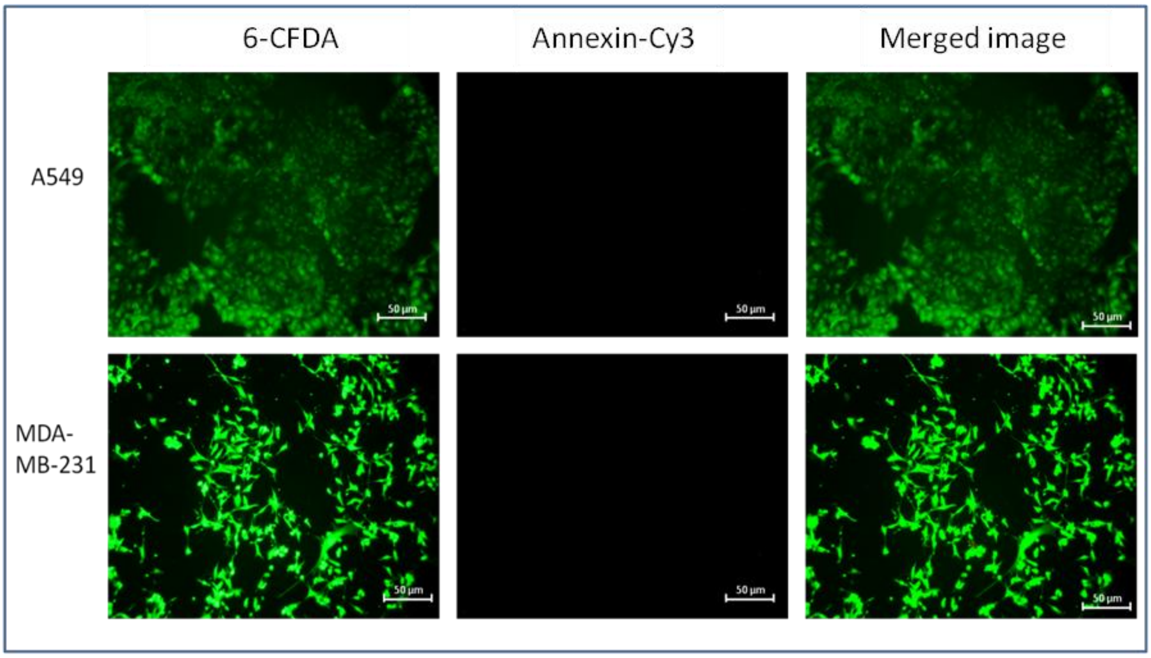

Figure S11: Images of A549 (upper panel) and MDA-MB-231 (lower panel) cells treated with the drug carrier, GP alone. The green fluorescent (left panel) represents the non-apoptotic cells. The middle panel will be stained red in the presence of necrotic cells. In the overlay images (right panel) the cells will emit yellowish-green fluorescence in the presence of early stages of apoptosis. The absence of red and yellowish-green stain in the images shows that the cells did not undergo apoptosis.
